# Nonoperative Management of Pediatric Liver Injury: Current Evidence, Clinical Indications, and Outcomes

**DOI:** 10.3390/medicina62061088

**Published:** 2026-06-04

**Authors:** Marius Dumitru Dănilă, Lavinia Țocu, Bogdan Ioan Ștefănescu, Florentin Dimofte, Valerii Luțenco, Loredana Stavăr Matei, Sorin Ion Berbece, Iulia Chiscop, Mădălina Nicoleta Matei, Paul Iacobescu, Victor Relu Savastre, George Țocu

**Affiliations:** 1 Faculty of Medicine and Pharmacy, Research Center in the Medical-Pharmaceutical Field, “Dunărea de Jos” University, 800008 Galati, Romania; marius.danila@ugal.ro (M.D.D.); florentin.dimofte@ugal.ro (F.D.); valerii.lutenco@ugal.ro (V.L.); loredana.matei@ugal.ro (L.S.M.); iulia.chiscop@ugal.ro (I.C.); madalina.matei@ugal.ro (M.N.M.); george.tocu@ugal.ro (G.Ț.); 2 “Sf. Ioan” Children’s Emergency Hospital, 800487 Galati, Romania; 3 “Sf. Apostol Andrei” County Emergency Clinical Hospital, 800578 Galati, Romania; sorin.berbece@ugal.ro; 4 Computer Science and Information Technology Department, “Dunărea de Jos” University, 800008 Galati, Romania; paul.iacobescu@ugal.ro; 5 Special Action Service of Police, 800446 Galati, Romania; svastrev@gmail.com

**Keywords:** pediatric liver injury, blunt abdominal trauma, nonoperative management, pediatric trauma, hepatic trauma

## Abstract

*Background and Objectives*: Pediatric liver injury is a frequent solid organ injury after blunt abdominal trauma, and its management has progressively shifted toward nonoperative care in hemodynamically stable children. This narrative review aims to synthesize current evidence regarding diagnosis, eligibility for nonoperative management, inpatient monitoring, outcomes, complications, escalation criteria, and post-discharge care in pediatric liver trauma. *Materials and Methods*: A structured literature search was performed in PubMed/MEDLINE, Scopus, and Web of Science, with supplementary screening through Google Scholar and reference lists. Publications from January 2000 to December 2025 were considered. The literature was analyzed descriptively and thematically, without formal risk-of-bias assessment, evidence grading, or quantitative meta-analysis. *Results*: The available evidence supports nonoperative management for most children with blunt liver injury who are hemodynamically stable or show a sustained response to initial resuscitation. Eligibility depends primarily on physiological status, clinical evolution, associated injuries, and institutional capability rather than imaging grade alone. Nonoperative management requires structured clinical, hemodynamic, and laboratory reassessment, with follow-up imaging reserved for selected cases based on clinical evolution or suspected complications. Delayed hemorrhage, bile leak, biloma, pseudoaneurysm, hemobilia, infection, and failure of nonoperative management remain clinically relevant and may require repeat imaging, interventional radiology, or surgery. *Conclusions*: Nonoperative management should be understood as an active organ-preserving strategy based on careful selection, serial reassessment, and immediate access to escalation when needed. Further pediatric liver-specific studies are required to standardize monitoring, repeat imaging, intervention thresholds, activity restriction, and post-discharge follow-up.

## 1. Introduction

Pediatric abdominal trauma remains an important cause of morbidity worldwide, with the liver representing one of the most frequently injured solid organs in children following blunt abdominal trauma [1,2]. Road traffic accidents, falls, bicycle-related injuries, sports trauma, and nonaccidental injury may all contribute to hepatic damage in the pediatric population, with clinical presentations ranging from minor parenchymal lesions to complex injuries associated with hemodynamic instability and multi-organ trauma [3]. Because of the unique anatomical and physiological characteristics of children, including greater chest wall compliance, less protective musculature, and a relatively larger solid organ volume in proportion to body size, the liver is particularly vulnerable to traumatic injury [4].

Over the past decades, the management of pediatric liver trauma has undergone a major paradigm shift. Historically, operative exploration was frequently performed in children with significant hepatic injury because of concerns regarding ongoing hemorrhage, missed associated lesions, and the limited availability of advanced imaging and intensive monitoring [5]. However, progressive improvements in trauma systems, pediatric critical care, transfusion practices, cross-sectional imaging, and interventional radiology have supported the increasing adoption of nonoperative management as the preferred strategy in hemodynamically stable patients [2,6]. This transition has paralleled broader changes in pediatric trauma care, where organ preservation, reduction in unnecessary laparotomies, and minimization of treatment-related morbidity have become central goals.

Nonoperative management is now widely accepted as the preferred strategy for most hemodynamically stable children with blunt liver injury, as well as for selected patients who show a sustained response to initial resuscitation. Its benefits include avoidance of surgical trauma, lower rates of postoperative complications, shorter recovery in appropriately selected cases, and preservation of hepatic tissue and function [2,6,7]. At the same time, successful nonoperative management requires careful patient selection, accurate injury assessment, close clinical monitoring, and timely recognition of complications or treatment failure. The decision to continue observation rather than proceed to operative or interventional treatment is therefore not based solely on imaging grade, but on a complex integration of physiological status, associated injuries, resource availability, and institutional expertise [8,9,10].

Despite the widespread adoption of nonoperative management, several aspects of pediatric liver trauma care remain incompletely standardized. The literature includes heterogeneous study populations, variable definitions of hemodynamic instability, differences in injury grading and follow-up protocols, and inconsistent criteria for angioembolization, repeat imaging, duration of hospitalization, and activity restriction after discharge. Because most available evidence is derived from observational cohorts, institutional pathways, and broader solid organ injury studies, these areas should be interpreted as unresolved clinical questions rather than as issues that can be definitively settled by the existing literature.

In this review, pediatric patients are defined as individuals younger than 18 years, consistent with commonly used pediatric trauma frameworks [7], while recognizing that trauma pathways may also be influenced by developmental stage, body size, institutional policy, and local pediatric trauma resources. Although selected principles, such as physiology-guided decision making and organ preservation, may be relevant to adolescents or selected young adults, pediatric algorithms should not be directly extrapolated to adults without considering physiological, anatomical, and organizational differences.

Although nonoperative management is now widely adopted for most hemodynamically stable children with blunt liver injury, its practical application remains shaped by predominantly observational and heterogeneous evidence, variable institutional resources, and differences in monitoring, follow-up, and escalation protocols. This narrative review therefore aims to provide a focused liver-specific synthesis of evidence derived from cohort studies, institutional experiences, clinical reviews, guidelines, and consensus documents, integrating patient selection, diagnostic assessment, inpatient monitoring, outcomes, complications, escalation to interventional radiology or surgery, and post-discharge care into a practical clinical framework.

## 2. Materials and Methods

This narrative review was conducted to provide a focused and clinically relevant synthesis of the literature on the nonoperative management of pediatric liver injury. The review was designed as a narrative review with a structured search strategy, but not as a systematic review or meta-analysis. Therefore, no review protocol was registered, no formal risk of bias assessment was performed, and Preferred Reporting Items for Systematic Reviews and Meta Analyses (PRISMA) methodology was not applied.

A structured literature search was performed in PubMed/MEDLINE, Scopus, and Web of Science, with supplementary screening through Google Scholar and the reference lists of relevant articles. The search covered publications from January 2000 to December 2025, with the final search update completed in December 2025. Only articles published in English were retained for analysis.

The search strategy combined keywords and relevant Medical Subject Headings (MeSH) related to pediatric liver injury and nonoperative management, including “pediatric liver trauma”, “hepatic injury in children”, “blunt abdominal trauma”, “pediatric solid organ injury”, “liver injury”, “nonoperative management”, and “angioembolization”. Boolean operators were used to refine the search, with combinations such as “pediatric liver trauma” AND “nonoperative management”; “hepatic injury in children” AND “nonoperative management”; “blunt abdominal trauma” AND “liver injury” AND “children”; and “pediatric liver trauma” AND “angioembolization”.

Eligible sources included original clinical studies, retrospective and prospective cohort studies, registry-based analyses, systematic reviews, narrative or clinical reviews, guideline papers, and consensus documents addressing pediatric hepatic trauma. Case reports were considered only when they described rare complications or management issues not adequately represented in larger studies. Studies focused exclusively on adult populations without pediatric applicability, nonclinical experimental data, unrelated trauma sites, or isolated surgical techniques without relevance to nonoperative management were excluded from the main synthesis.

Following database and supplementary searches, a total of 853 records were initially identified. After the removal of 248 duplicates, 605 records were screened by title and abstract. Of these, 155 records were excluded because they were not relevant to pediatric liver trauma or did not focus on clinical management. The remaining 450 full-text publications were reviewed for potential inclusion, and 373 were not retained because they focused mainly on adult populations without pediatric applicability, experimental or nonclinical topics, unrelated trauma sites, isolated surgical techniques, nonrelevant case reports, or publications in languages other than English, *n* = 22. Finally, 77 references were retained and cited in the narrative synthesis. Based on source type, these included 50 original clinical or epidemiological studies, 3 systematic reviews, scoping reviews, or meta-analyses, 17 narrative or clinical reviews and book chapters, 7 guideline, consensus, classification, or manual-based documents, and no standalone case reports.

The selection of studies was based on clinical relevance, methodological clarity, pediatric applicability, and contribution to the main objectives of the review. Priority was given to publications reporting quantitative data on success rates, failure of nonoperative management, complications, hospital stay, transfusion needs, interventional radiology, surgery, morbidity, mortality, or post-discharge follow-up. The available literature was analyzed descriptively and thematically, with emphasis on patient selection, diagnostic workup, inpatient monitoring, outcomes, complications, escalation criteria, and outpatient management.

## 3. Epidemiology, Mechanisms, and Injury Grading

A clear understanding of the epidemiology, injury mechanisms, and grading systems of pediatric liver trauma is essential for placing nonoperative management into its proper clinical and pathophysiological context. These aspects help define both the burden and the complexity of hepatic injury in children and provide the framework for subsequent diagnostic and therapeutic decisions. This section outlines the main epidemiological, mechanistic, and classificatory elements relevant to pediatric liver trauma.

### 3.1. Epidemiology of Pediatric Liver Injury

Liver injury is one of the most common solid organ injuries in children with blunt abdominal trauma. Together with the spleen, the liver accounts for a substantial proportion of pediatric abdominal visceral injuries and represents a frequent indication for hospital admission, imaging evaluation, and trauma monitoring [11,12]. In a retrospective study of 246 children with blunt abdominal trauma, Zakaria et al. reported splenic injury in 153 patients, 62%, liver injury in 78 patients, 32%, and combined liver and spleen injury in 15 patients, 6% [12]. The clinical importance of pediatric liver injury is related not only to incidence, but also to the wide spectrum of lesions, ranging from minor subcapsular hematomas to complex parenchymal disruption associated with major hemorrhage or concomitant injuries [11,12].

The epidemiology of pediatric liver trauma is closely linked to the general pattern of childhood injury. In younger children, domestic accidents and falls are more frequent mechanisms, whereas in older children and adolescents, road traffic accidents, bicycle collisions, pedestrian injuries, and sports-related trauma become increasingly relevant. Non-accidental trauma should also be specifically considered, particularly in infants and younger children or when the reported mechanism is inconsistent with the injury pattern. Recognition of possible abuse is essential because it changes not only the diagnostic workup, but also safeguarding, documentation, and multidisciplinary management [4]. The relative contribution of each mechanism varies by geographic region, socioeconomic context, and access to preventive safety measures such as seat belts, child restraints, and helmet use. Seasonal and environmental factors may also influence injury patterns, particularly in settings where recreational outdoor activities are frequent [13,14].

From a public health perspective, pediatric liver injury deserves attention because trauma remains a leading cause of death and disability in children worldwide [15]. Although many hepatic injuries are managed without surgery, they still require rapid assessment, institutional resources, imaging access, and experienced multidisciplinary care. The burden of these injuries extends beyond the acute phase, as hospitalization, caregiver anxiety, school absence, temporary activity restriction, and post-traumatic follow-up may all contribute to the broader clinical and social impact. Therefore, understanding the epidemiologic profile of pediatric liver trauma is important not only for clinical decision making, but also for prevention strategies and resource allocation in pediatric trauma systems.

### 3.2. Mechanisms of Trauma and Patterns of Hepatic Injury

In pediatric patients, blunt trauma is the predominant mechanism of liver injury [16,17]. Hepatic lesions may result from direct compression, deceleration, or a combination of both. Compression forces can produce superficial lacerations, contusions, or subcapsular hematomas, whereas deceleration trauma may lead to deeper parenchymal tears and vascular injury. Depending on trauma severity, liver injury may occur in isolation or in association with thoracic, splenic, renal, intestinal, or cranial lesions [17].

The pattern of hepatic damage is influenced by both the intensity and direction of the traumatic force. Direct blows to the upper abdomen or lower thorax may compress the liver against the spine or rib cage, while sudden deceleration may generate shearing stress at vascular pedicles or ligamentous fixation points [18]. These mechanisms explain why liver trauma may range from focal peripheral lesions to more extensive injuries involving central parenchyma, hepatic veins, or major intrahepatic vessels. In some cases, biliary disruption may occur either as an immediate consequence of trauma or as a delayed manifestation.

In clinical practice, the distinction between isolated and polytrauma-related liver injury is highly relevant. Children with isolated hepatic trauma are often more suitable for nonoperative management and may have a more predictable clinical course than those with multiple associated injuries [17,19]. By contrast, children with extra-abdominal trauma or multiple intra-abdominal injuries require broader diagnostic evaluation and may have more complex therapeutic priorities. In addition, the mechanism of injury may itself raise suspicion for occult lesions. High-energy trauma, particularly in motor vehicle collisions, should prompt careful assessment for combined abdominal and thoracic damage, even when the initial presentation appears relatively stable [1,17].

### 3.3. Pediatric Anatomical and Physiological Particularities

Children have several anatomical and physiological characteristics that increase susceptibility to liver trauma. The thoracic cage is more compliant, the abdominal wall musculature is less developed, and the liver occupies a relatively larger volume in proportion to body size than in adults. As a result, traumatic forces are transmitted more easily to the hepatic parenchyma. In addition, external signs of trauma may be limited even in the presence of clinically significant intra-abdominal injury, which makes careful clinical assessment and appropriate imaging essential during the initial evaluation. Developmental age further complicates assessment, as younger children may be unable to localize pain, describe symptoms reliably, or communicate clinical worsening, making repeated observation and caregiver input particularly important [1,2].

These pediatric-specific features also influence the clinical expression of injury. Children may maintain blood pressure despite significant blood loss through compensatory vasoconstriction and tachycardia, so early hemodynamic deterioration can be subtle. Reliance on blood pressure alone may delay recognition of clinically important hemorrhage [20]. For this reason, serial assessment should integrate heart rate, capillary refill, mental status, skin perfusion, urine output, laboratory trends, and response to resuscitation.

The hepatic tissue itself may also respond differently to trauma in children compared with adults. The relative elasticity of tissues and the absence of preexisting chronic liver disease in most pediatric patients may support recovery when bleeding is controlled and perfusion is maintained. At the same time, the limited circulating blood volume of children increases the clinical significance of even moderate hemorrhage [20]. Consequently, the apparent size or grade of the lesion on imaging does not always reflect the full physiological impact on the child [21]. These anatomical and physiological particularities support individualized assessment and reinforce the need for pediatric-adapted trauma protocols rather than direct extrapolation from adult practice.

### 3.4. Classification Systems and Injury Grading

The severity of hepatic trauma is most commonly described using the American Association for the Surgery of Trauma (AAST) liver injury scale, updated in 2018. The current AAST organ injury scale classifies liver injuries from grade I to grade V according to imaging, operative, and pathological criteria, including hematoma extent, laceration depth, vascular injury, and hepatic devascularization [21]. In clinical practice, lower-grade injuries generally include minor hematomas and superficial lacerations, whereas high-grade lesions are characterized by extensive parenchymal disruption, major vascular involvement, or severe tissue destruction. The grading system facilitates standardized reporting, comparison among studies and trauma centers, and communication within multidisciplinary trauma teams [8,22].

Radiologic grading is usually based on Computed Tomography (CT) findings in hemodynamically stable or stabilized patients. CT provides detailed information about lesion depth, the extent of subcapsular or intraparenchymal hematoma, active contrast extravasation, and associated abdominal injuries, making it central to the anatomic characterization of pediatric liver trauma [23]. In contrast, ultrasonography, including Focused Assessment with Sonography for Trauma (FAST), is useful as an initial screening tool, but it is less precise for accurate grading and for identifying the full extent of parenchymal injury [24].

Beyond its descriptive role, injury grading has significant value in research and communication. It allows clinicians to stratify cases, compare institutional outcomes, and evaluate whether management outcomes vary according to lesion severity. It also helps frame expectations regarding possible complications such as delayed bleeding, biliary collections, or pseudoaneurysm formation. Nevertheless, grading systems are not without limitations. Interobserver variability, differences in imaging timing, and the evolving nature of traumatic lesions may all affect classification accuracy. As a result, injury grade should be interpreted as an important structural parameter that supports risk stratification and comparison between cohorts, but should not replace integrated clinical assessment [25].

### 3.5. Clinical Relevance of Injury Severity Classification

Although injury grading remains important for radiologic description and severity stratification, it should not function as an isolated determinant of management. Pediatric solid organ injury guidelines and cohort data emphasize the need to integrate hemodynamic status, response to resuscitation, associated injuries, and evidence of ongoing bleeding when choosing between observation, interventional radiology, or surgery [8,26]. Therefore, injury classification is clinically useful when interpreted within the broader physiological and injury context, rather than as a stand-alone therapeutic guide.

This principle is particularly important in pediatric liver trauma, where selected high-grade injuries may still be managed nonoperatively when the child remains hemodynamically stable and adequate monitoring resources are available. Conversely, lower-grade lesions may require escalation when accompanied by persistent hemodynamic compromise, associated bowel injury, worsening abdominal findings, or evidence of active hemorrhage [27]. Thus, the same anatomic grade may have different management implications depending on the overall physiological and clinical context.

From a practical standpoint, injury severity classification is most useful when it supports, rather than replaces, clinical judgment. It contributes to risk stratification, informs surveillance intensity, and may help determine whether interventional radiology consultation or transfer to a higher-level trauma center should be considered. It also provides a common framework for communication within the multidisciplinary team and with the family. However, pediatric solid organ injury recommendations emphasize that continued observation should be guided primarily by repeated clinical and laboratory reassessment, with repeat imaging reserved for selected cases when clinical evolution or suspected complications justify it, rather than by the initial CT grade alone [28].

## 4. Initial Assessment and Diagnostic Workup

**Figure 1:**
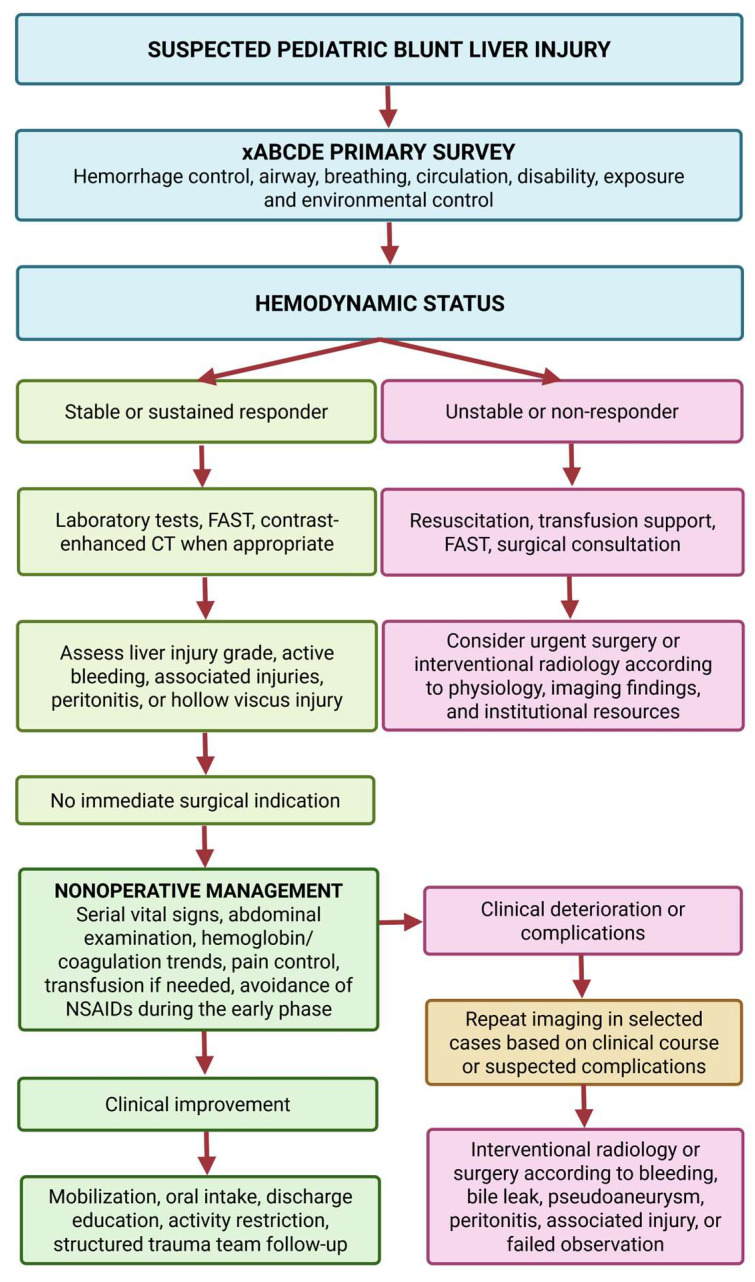
Proposed Assessment and Management Algorithm for Pediatric Blunt Liver Injury. This figure summarizes a proposed clinical pathway for suspected pediatric blunt liver injury, developed by the authors based on the reviewed literature and current pediatric trauma management principles. The algorithm begins with the xABCDE primary survey, including immediate control of exsanguinating hemorrhage when present, followed by assessment of airway, breathing, circulation, disability, exposure and environmental control, vital signs, and physical examination. Hemodynamic status guides the early pathway. Stable patients or those with a sustained response to resuscitation undergo laboratory testing, FAST, and contrast-enhanced CT when appropriate, followed by evaluation of injury grade, active bleeding, and associated lesions. Patients without immediate indications for surgery may proceed to nonoperative management with structured clinical, hemodynamic, and laboratory reassessment, pain control, transfusion if needed, and avoidance of nonsteroidal anti-inflammatory drugs (NSAIDs) during the early phase. Repeat imaging should be reserved for selected cases based on the clinical course or suspected complications. Escalation to interventional radiology or surgery should be guided by patient physiology, local expertise, and the suspected mechanism of failure, including hemorrhagic, biliary, vascular, or peritoneal complications, as well as complications related to associated injuries.

The initial evaluation of pediatric liver injury is a critical step in determining trauma severity, identifying associated lesions, and guiding subsequent therapeutic decisions. Early clinical assessment, laboratory testing, and imaging must be integrated carefully in order to support timely decision making and avoid both missed injuries and unnecessary interventions. This section presents the main components of the initial assessment and diagnostic workup in pediatric hepatic trauma, which represent the entry point for the proposed assessment and management pathway summarized in Figure 1.

### 4.1. Primary Survey and Hemodynamic Assessment

The initial evaluation of pediatric liver trauma begins with a structured primary survey according to the xABCDE approach described in the 11th edition of Advanced Trauma Life Support (ATLS), including immediate control of exsanguinating hemorrhage when present, followed by airway, breathing, circulation, disability, exposure, and environmental control assessment [29]. Although hepatic injury is an intra-abdominal lesion, the first priority remains the identification and treatment of immediately life-threatening conditions, particularly airway compromise, respiratory distress, tension pneumothorax, or massive hemorrhage. In many pediatric trauma cases, liver injury occurs in the context of multisystem trauma, and the clinician must therefore avoid narrowing the assessment prematurely to the abdomen alone [30,31].

Hemodynamic evaluation is essential during the primary survey. In pediatric patients, circulatory status requires careful interpretation because children can maintain arterial pressure despite significant blood loss through compensatory tachycardia and peripheral vasoconstriction. For this reason, hypotension is often a late and concerning sign of decompensation [32]. Early assessment should integrate heart rate, capillary refill, pulse quality, mental status, skin perfusion, urine output, and response to initial fluid or blood resuscitation. Initial classification as stable, transient responder, or persistently unstable should be documented because it guides the urgency of diagnostic workup, observation, or intervention [33].

Clinical judgment during the primary survey must also take into account the mechanism of injury. High-energy trauma, such as road traffic accidents or significant falls, increases the likelihood of major intra-abdominal injury, even when the child appears initially stable. Similarly, associated thoracic trauma, altered consciousness, or distracting orthopedic injuries may obscure abdominal symptoms [4,34]. Therefore, early hemodynamic assessment in pediatric liver trauma should not be viewed as a single momentary evaluation, but rather as a dynamic process that continues throughout resuscitation and early inpatient care.

### 4.2. Clinical Presentation and Physical Examination

The clinical presentation of pediatric liver injury is variable and may range from mild abdominal discomfort to overt signs of hemorrhagic shock. Children may present with right upper quadrant pain, diffuse abdominal tenderness, abdominal distension, guarding, pallor, tachycardia, or decreased activity level. In some cases, referred pain to the right shoulder may occur because of diaphragmatic irritation, although this is less consistently reported in younger children. Nausea, vomiting, agitation, or lethargy may also be present, particularly in the setting of significant trauma or concomitant injuries [35].

Physical examination remains a key component of the assessment, but it has important limitations. Localized tenderness, abdominal wall bruising, seatbelt marks, or signs of peritoneal irritation may raise suspicion for intra-abdominal injury, yet their absence does not exclude significant hepatic trauma. This is especially relevant in children, in whom external signs may be minimal despite substantial internal damage. Furthermore, pain may be difficult to localize in younger patients, and examination findings may be altered by fear, crying, sedation, or associated head injury. For these reasons, the abdominal examination should be repeated serially rather than relied upon as a one-time determinant [4,36].

A careful secondary survey is also important for identifying associated lesions that may influence management priorities. Thoracic injuries, pelvic trauma, long bone fractures, and neurologic impairment may coexist and significantly affect both the reliability of physical examination and the overall management plan [30,37]. Thus, clinical findings provide valuable clues, but they should be interpreted within the broader trauma context and in conjunction with laboratory and imaging data.

### 4.3. Laboratory Investigations

Laboratory studies play a supportive but important role in the diagnostic workup of pediatric liver injury and are useful for establishing a baseline, monitoring possible ongoing blood loss, and detecting associated physiological disturbances. Initial testing commonly includes hemoglobin or hematocrit, blood type and crossmatch, coagulation profile, liver enzymes, serum lactate, electrolytes, renal function markers, and blood gas analysis when clinically indicated. These investigations do not replace imaging, but they provide complementary information regarding trauma severity, systemic response to injury, perfusion status, and the need for repeated reassessment [1,38].

Hemoglobin values may be normal at presentation, especially in the early phase after acute hemorrhage, and should therefore be interpreted with caution. Serial measurements are more informative than isolated values, particularly when correlated with hemodynamic trends and clinical findings [1,38]. Elevated aminotransferases may support suspicion of hepatic injury in blunt abdominal trauma, although they are not specific and do not independently determine the extent of parenchymal damage. In clinically equivocal cases, markedly increased transaminase levels may help identify children who warrant further imaging evaluation, particularly when physical signs are subtle [39].

Coagulation abnormalities may reflect hemorrhage, shock, dilution after resuscitation, or traumatic coagulopathy, all of which are relevant to risk stratification and management planning. Lactate and acid-base status may provide additional insight into tissue perfusion and the adequacy of resuscitation [40]. Overall, laboratory evaluation in pediatric liver trauma should be interpreted as part of an integrated clinical, hemodynamic, and imaging assessment. Its greatest value lies in serial monitoring and in supporting management decisions, rather than in establishing the diagnosis in isolation [8,40].

### 4.4. Imaging Approach, FAST and CT

Imaging is fundamental to the diagnosis and characterization of pediatric liver injury. FAST is widely used as an early bedside tool for detecting intraperitoneal free fluid in children with abdominal trauma. It offers several practical advantages, including rapid performance, absence of ionizing radiation, repeatability, and usefulness during the initial resuscitative phase [1,24]. In unstable patients, the detection of free fluid may support urgent decision-making, particularly when correlated with hemodynamic compromise and mechanism of injury [24].

However, FAST has important limitations in pediatric hepatic trauma. It may miss small volumes of intraperitoneal fluid, does not characterize the exact parenchymal lesion, and cannot reliably grade liver injury or identify all associated abdominal injuries. A negative FAST examination does not exclude clinically significant intra-abdominal trauma, especially in children who remain symptomatic or who were exposed to high-energy mechanisms. Pediatric data therefore support using FAST as an adjunct to the initial assessment, rather than as a definitive diagnostic tool [41].

Contrast-enhanced CT remains the reference imaging modality for hemodynamically stable or stabilized children with suspected liver injury. It allows accurate localization and grading of hepatic lesions, identification of active contrast extravasation, evaluation of subcapsular and intraparenchymal hematomas, and detection of associated abdominal and retroperitoneal injuries [24,42]. CT also provides essential information for planning observation, interventional radiology, or surgery when needed. Nevertheless, it must be used judiciously because of radiation exposure, particularly in the pediatric population. The decision to perform CT should therefore balance diagnostic yield, clinical necessity, and the need to minimize unnecessary imaging [42].

### 4.5. Diagnostic Challenges and Differential Considerations

The diagnostic evaluation of pediatric liver trauma is often complicated by nonspecific symptoms, variable reliability of physical examination, and the frequent coexistence of other traumatic lesions. Younger children may have difficulty describing pain, while altered mental status, sedation, or distracting injuries may further limit clinical assessment. As a result, diagnosis requires a high index of suspicion, particularly when the trauma mechanism is significant or when early hemodynamic findings are borderline [1,40].

An additional challenge lies in distinguishing isolated hepatic injury from broader intra-abdominal trauma. Splenic lesions, renal injuries, bowel perforation, pancreatic trauma, and mesenteric injury may coexist and substantially alter management priorities. Free intraperitoneal fluid without an immediately obvious solid organ injury should raise concern for hollow viscus or mesenteric injury, particularly when abdominal findings progress or inflammatory markers worsen [43,44]. In this context, the diagnostic workup should remain sufficiently broad to avoid focusing exclusively on the liver once an initial hepatic lesion has been identified.

Nontraumatic or preexisting hepatic conditions rarely mimic acute traumatic injury, but they may occasionally complicate interpretation when imaging findings are atypical. Delayed manifestations such as biliary leakage, pseudoaneurysm formation, or secondary hemorrhage may also be absent at the time of first evaluation. For this reason, the diagnostic process should not be considered complete after the initial CT scan. New symptoms, falling hemoglobin levels, persistent tachycardia, fever, jaundice, or worsening abdominal findings should prompt diagnostic reconsideration, repeat imaging when indicated, and reassessment of the need for interventional radiology or surgery [8,44].

## 5. Indications and Principles of Nonoperative Management

The growing role of nonoperative management in pediatric liver trauma reflects both advances in trauma care and a better understanding of which children can be treated safely without surgery. Appropriate patient selection, careful interpretation of hemodynamic status, and adherence to core management principles remain central to the success of this approach. This section summarizes the main indications and guiding principles of nonoperative management in pediatric hepatic injury.

### 5.1. Patient Selection for Nonoperative Management

Appropriate patient selection is the cornerstone of successful nonoperative management in pediatric liver injury. Although hepatic trauma ranges from minor capsular lesions to severe parenchymal disruption, radiologic severity alone does not mandate operative intervention. Current pediatric solid organ injury literature and liver trauma guidelines support nonoperative management in selected high-grade injuries when the child is hemodynamically stable or shows a sustained response to resuscitation, provided that serial monitoring, pediatric surgical expertise, imaging, transfusion support, and timely escalation options are available [2,8].

The selection process begins with an integrated assessment of trauma mechanism, vital signs, physical findings, laboratory trends, and imaging results. Children who remain clinically stable after initial evaluation are generally eligible for nonoperative management. Equally important is the absence of findings that mandate laparotomy independently of the hepatic lesion, such as hollow viscus perforation, diffuse peritonitis, or uncontrolled intra-abdominal contamination. Guideline-based recommendations and pediatric cohort data indicate that selection for nonoperative management should integrate hemodynamic status, associated injuries, and institutional capability, rather than relying on the liver lesion considered in isolation [28,45].

Age, comorbid conditions, associated trauma, and institutional resources may also influence selection. Younger children may require closer observation because clinical deterioration can be subtle or more difficult to interpret. Children with polytrauma, severe thoracic injury, traumatic brain injury, or coagulation abnormalities often require a more individualized approach. Clinical series of pediatric blunt hepatic trauma have also supported nonoperative management in stable children without immediate operative indications [46]. Therefore, patient selection should be viewed as a dynamic process of risk stratification rather than a rigid checklist.

### 5.2. Hemodynamic Stability as the Key Determinant

Among all factors that influence management strategy, hemodynamic stability remains the most important determinant of eligibility for nonoperative management. In contemporary pediatric trauma care, the primary question is not simply how severe the liver injury appears on imaging, but whether the child is able to maintain adequate perfusion without evidence of ongoing uncontrolled hemorrhage [2,8]. This principle is supported by pediatric solid organ injury data. In the study by McVay et al., 101 children with isolated spleen or liver injury were managed using a pathway based on hemodynamic status, including 51 children with liver injury. The mean actual length of stay was 1.9 days compared with 3.2 projected days according to grade based American Pediatric Surgical Association guidelines, and for grade III to V injuries it was 2.5 versus 4.3 days, respectively. All patients in this cohort returned to full activity without complications [45]. These findings reinforce the practical value of physiology guided management in selected pediatric liver and spleen injuries.

Hemodynamic stability in children must be interpreted carefully and in a manner adapted to age specific physiology. A child may initially maintain blood pressure despite substantial blood loss through tachycardia and peripheral vasoconstriction. Consequently, the absence of hypotension should not lead to false reassurance. The clinician must instead consider a broader set of indicators, including heart rate trends, capillary refill, mental status, peripheral perfusion, urine output, and response to resuscitation. Children who respond promptly and durably to initial fluid or blood administration may still be candidates for observation, while those with persistent or recurrent instability require a lower threshold for escalation [20,47].

The practical importance of hemodynamic status lies in its ability to guide triage and timing. A stable child can undergo a more complete diagnostic workup, including contrast-enhanced CT, and can generally be monitored within a structured nonoperative pathway. By contrast, a child with worsening shock despite resuscitation requires immediate reassessment and may need urgent intervention, either by surgery or, in selected settings, by interventional radiology.

### 5.3. Indications and Contraindications

The principal indication for nonoperative management is blunt hepatic trauma in a child who is hemodynamically stable or who shows a sustained response to initial resuscitation, without evidence of another abdominal injury requiring emergency laparotomy. Guideline-based recommendations and pediatric cohort data support this approach across a wide range of injury grades, provided that the child can be monitored safely and that surgical, radiologic, transfusion, and intensive care support are available when needed [2,28,45]. Therefore, injury grade should be interpreted together with hemodynamic status, associated injuries, and institutional capability rather than used as an isolated indication for operative treatment.

Additional factors favoring nonoperative management include the absence of diffuse peritoneal irritation, the lack of radiologic evidence strongly suggestive of bowel perforation, and the availability of serial clinical assessment. Children with hepatic lacerations, subcapsular hematomas, or selected more extensive injuries may be managed safely under close observation when transfusion support, pediatric intensive care, and rapid access to intervention are available if needed. Pediatric cohort data and trauma guidelines support selective nonoperative management as a strategy that can preserve hepatic tissue and avoid laparotomy-related morbidity in appropriately selected patients [45,48]. This approach also aligns with the broader pediatric principle of minimizing invasive procedures when safe alternatives exist.

Contraindications to continued observation are usually determined by the overall clinical picture rather than by liver injury grade alone. Persistent hemodynamic instability despite resuscitation remains the clearest reason to avoid or abandon nonoperative management and proceed to urgent intervention. Other important triggers for escalation include suspected hollow viscus injury, diffuse or worsening peritonitis, uncontrolled transfusion requirement, or evidence of ongoing hemorrhage that cannot be safely managed by observation, transfusion support, or timely interventional radiology. In addition, nonoperative management may not be appropriate in hospitals lacking adequate pediatric monitoring, imaging availability, blood bank support, or timely access to surgery and interventional radiology. Pediatric guidelines and cohort-based evidence emphasize that indications for observation, intervention, or transfer should be interpreted within the clinical and organizational context in which the child is treated [28,45,48].

### 5.4. Core Principles of Nonoperative Management

Nonoperative management of pediatric liver injury is not a passive decision to avoid surgery, but an active and structured therapeutic strategy based on repeated reassessment and anticipatory care. Its core principles include hemodynamic surveillance, serial abdominal evaluation, appropriate laboratory follow-up, judicious transfusion support, pain control, and timely identification of changes suggesting failure of nonoperative management. Updated pediatric guidance emphasizes structured monitoring and management pathways adapted to the child’s physiological status and clinical evolution [49].

A central principle is that observation must be responsive rather than static. Children selected for nonoperative management require a clear monitoring plan tailored to injury severity and overall condition. This includes scheduled reassessment of vital signs, abdominal examination, hemoglobin trends, fluid balance, and signs of occult bleeding or evolving complications. Supportive care should be individualized and may include intravenous fluids, transfusion support when indicated, analgesia, antiemetics, and gradual nutritional progression according to tolerance and clinical evolution. Bed rest is often used in the early phase, although rigid and prolonged immobilization is increasingly questioned; in a prospective study of children with isolated spleen or liver injury, guideline-based care was used to standardize resource utilization and activity restriction rather than applying prolonged immobilization indiscriminately [19].

Another essential principle is that the goal of nonoperative management is not merely survival without surgery, but safe organ-preserving recovery. This requires a low threshold for reassessment if the clinical course changes. Falling hemoglobin, persistent tachycardia, increasing abdominal pain, abdominal distension, fever, or new biochemical abnormalities may indicate delayed bleeding, biliary complications, or associated lesions that were not initially apparent. Guideline-based recommendations and observational data emphasize structured monitoring, repeated reassessment, and timely escalation when the clinical trajectory becomes unfavorable [48,49,50].

### 5.5. Institutional Requirements and Multidisciplinary Care

The feasibility and safety of nonoperative management depend not only on patient factors, but also on the capabilities of the treating institution. Pediatric blunt abdominal trauma reviews, pediatric solid organ injury literature, and liver trauma guidelines emphasize that nonoperative management should be undertaken in centers able to provide continuous pediatric trauma observation, rapid imaging access, transfusion support, and immediate consultation with surgery, anesthesia, intensive care, diagnostic radiology, and interventional radiology teams [1,2,8]. In this context, nonoperative management should be regarded as an organized institutional pathway, not merely as an individual decision to avoid laparotomy.

A multidisciplinary model is particularly important in moderate and severe injuries. Emergency physicians, pediatric surgeons, trauma surgeons, radiologists, anesthesiologists, intensivists, and nursing staff all contribute to safe nonoperative management and timely escalation. Diagnostic radiologists are essential for accurate lesion characterization and for recognizing findings that may alter management, such as active contrast extravasation or biliary complications, while interventional radiology teams are central when angiography, embolization, or image-guided drainage is required [51]. Intensive care specialists support the management of children who require close hemodynamic monitoring, transfusion support, respiratory assistance, or management of multisystem trauma. Surgeons remain central even when no operation is performed, because continuation of nonoperative management requires ongoing surgical judgment.

Institutional readiness also determines whether escalation options are available when observation becomes insufficient. A hospital that cannot provide urgent laparotomy, pediatric intensive care, or interventional radiology may initiate stabilization, but transfer to a higher-level trauma center should be considered when the child is stable enough for transport and the anticipated benefit is substantial. Guideline-based recommendations and pediatric trauma pathways support interpretation of nonoperative management within the resources and response capacity of the treating institution [48,49,51]. This systems perspective is essential in pediatric liver trauma, where patient selection, monitoring quality, and rapid access to escalation all contribute to safe organ-preserving care.

## 6. Monitoring and In-Hospital Management

Once a nonoperative management pathway has been adopted, close monitoring and well-structured in-hospital care become essential for maintaining patient safety and supporting recovery. The safety of this approach depends not only on the initial decision to avoid surgery, but also on structured reassessment, appropriate supportive care, and timely recognition of clinical deterioration. This section highlights the main aspects of monitoring and inpatient management in pediatric liver trauma.

### 6.1. Clinical and Hemodynamic Monitoring

Careful in-hospital monitoring is essential after the decision to pursue nonoperative management in pediatric liver injury. The child should be observed within a structured clinical framework that allows early recognition of deterioration, delayed bleeding, or evolving complications. Monitoring intensity should be individualized according to injury severity, mechanism of trauma, initial hemodynamic profile, associated lesions, and response during the first hours after admission. Updated pediatric guidance and observational data support adapting the level of observation to the child’s clinical trajectory, with less intensive monitoring for minor isolated injuries and closer surveillance, including high dependency or intensive care settings when appropriate, for high-grade injuries, polytrauma, recent instability, or ongoing transfusion needs [49,52].

Hemodynamic monitoring remains a key component of inpatient observation. Serial recording of heart rate, blood pressure, respiratory rate, oxygen saturation, temperature, urine output, and peripheral perfusion is necessary to detect early signs of ongoing bleeding or inadequate resuscitation. In pediatric patients, heart rate trends may be more informative than isolated blood pressure values, because children can maintain arterial pressure until late in the course of significant blood loss. Clinical reassessment should therefore focus not only on numerical vital sign thresholds, but also on the overall trajectory of the child, including capillary refill, mental status, skin temperature, evolving fatigue, irritability, and response to supportive care [2,8,52].

Repeated abdominal examination is equally important, particularly during the first 24 to 48 h. Increasing pain, progressive distension, new guarding, or worsening tenderness may indicate bleeding, biliary leakage, or an associated intra-abdominal injury that was not initially evident. Because physical findings may evolve over time, serial examination by experienced clinicians remains an indispensable complement to laboratory and imaging follow-up. Observational pediatric data support continued clinical reassessment rather than reliance on a one-time confirmation of stability [53].

### 6.2. Laboratory Follow-Up and Transfusion Needs

Laboratory monitoring plays a supportive but important role in the inpatient management of pediatric liver injury. Serial hemoglobin and hematocrit measurements are commonly used to assess the risk of ongoing hemorrhage and to complement the clinical evaluation. However, these values should not be interpreted in isolation, as early measurements may remain deceptively normal after acute blood loss. In the ATOMAC+ secondary analysis by Stottlemyre et al., 767 children with blunt liver and/or spleen injury were included in one or more study cohorts; among 131 non-bleeding patients, hemoglobin decreased by a mean of 0.83 g/dL after a median of 6.3 h, showing that hemoglobin decline may occur even without intraperitoneal bleeding [52]. Therefore, the pattern of change over time should always be correlated with hemodynamic status, abdominal findings, and resuscitation context.

Liver enzymes may be useful at baseline and in selected follow-up situations, particularly when the clinical course raises concern for ongoing hepatic injury, biliary complications, or associated abdominal trauma. Coagulation studies are also relevant, especially in children with significant trauma, transfusion requirements, shock, or suspected traumatic coagulopathy. In more severe cases, lactate, acid-base status, renal function, and electrolyte trends may contribute to the broader evaluation of perfusion, resuscitation adequacy, and systemic physiological stress. Laboratory variables may also support risk stratification when interpreted together with clinical and hemodynamic findings, but routine repetition should remain clinically justified and adapted to the stability of the child rather than performed indiscriminately [54].

Transfusion decisions must be individualized and guided by both clinical and laboratory data. A falling hemoglobin concentration in a child who remains well perfused and clinically stable does not automatically mandate transfusion, whereas modest laboratory changes in the context of persistent tachycardia, worsening perfusion, coagulopathy, or suspected ongoing blood loss may justify escalation of transfusion and resuscitative support. Transfusion support should therefore be integrated into the overall resuscitation strategy rather than triggered by a single laboratory threshold. In a retrospective series of 47 children with blunt liver injury, Uysal and Aslan reported increasing transfusion requirements with higher injury grade, including transfusion in 13 patients with grade III injury and 8 patients with grade IV injury, while all 39 children managed nonoperatively survived [55]. During observation for pediatric liver injury, increasing transfusion requirements should therefore prompt reassessment of hemodynamic trajectory, coagulation status, and the need for interventional radiology or surgery [38,55].

### 6.3. Bed Rest, Mobilization, and Supportive Care

Supportive care is a major component of nonoperative management and extends well beyond simple observation. During the early post-traumatic period, bed rest is commonly recommended, particularly in children with moderate or severe injuries, active symptoms, or recent hemodynamic instability. The rationale is to reduce physical stress during the vulnerable phase of hemorrhage control and tissue stabilization. However, contemporary practice increasingly favors an individualized approach rather than prolonged and uniform immobilization. Pediatric guidance supports mobilization according to clinical stability rather than injury grade alone [49]. In a prospective pediatric study of blunt spleen and liver injury, St Peter et al. used an abbreviated protocol with 1 night of bed rest for grade I to II injuries and 2 nights for grade III or higher injuries, with no readmissions reported [56].

Pain management should be effective and clinically judicious, as inadequate analgesia may hinder serial examination, reduce respiratory effort, and contribute to anxiety or poor cooperation. Analgesic strategies should relieve discomfort while avoiding oversedation that might obscure clinical deterioration. During the early phase, medications with anticoagulant or antiplatelet effects, particularly nonsteroidal anti-inflammatory drugs (NSAIDs), should generally be avoided unless specifically justified by the treating team. Additional supportive measures include fluid management, antiemetics when needed, nutritional progression according to tolerance, and maintenance of reliable venous access in case rapid intervention becomes necessary. In children with nausea, abdominal discomfort, or concern for associated injuries, oral intake may need to be delayed briefly, but prolonged fasting is generally unnecessary once the clinical condition stabilizes [56,57].

Nursing care is particularly important during inpatient nonoperative management. Frequent reassessment, accurate recording of intake and output, early recognition of behavioral changes, and prompt reporting of evolving pain or abdominal findings contribute to early detection of clinical deterioration. Emotional support for both the child and caregivers should not be overlooked, as trauma-related distress may complicate cooperation and recovery. Pediatric inpatient care pathways emphasize the importance of structured observation, documentation, and communication within the multidisciplinary team [58].

### 6.4. Intensive Care Considerations

Not all children with hepatic trauma require admission to a pediatric intensive care unit, but higher levels of monitoring are appropriate in selected cases. Intensive care is particularly relevant for patients with high-grade liver injuries, recent hemodynamic instability, substantial transfusion needs, associated thoracic or neurologic trauma, or a clinical trajectory that remains uncertain during the first hours after admission. In a National Trauma Data Bank analysis of 5777 children with isolated blunt liver and spleen injuries, Wang et al. identified 2031 low-risk patients, 35.2%, and reported that ICU admission still occurred in 30.9% of low-risk patients compared with 41.6% of high-risk patients, suggesting that ICU use should be individualized according to clinical risk rather than applied routinely [10]. In higher-risk patients, nonoperative management remains possible, but it requires a setting where deterioration can be detected immediately and escalation can occur without delay [49].

The role of intensive care is not limited to hemodynamic observation. Children admitted to the ICU may require advanced respiratory support, invasive or frequent monitoring, management of coagulopathy, strict fluid balance surveillance, or coordinated treatment of multisystem trauma. In a multicenter retrospective study of 1401 children with blunt liver and spleen injury, Yamamoto et al. reported that 421 patients were treated in hospitals with PICUs and 207 were admitted directly to a PICU; adjusted 30-day mortality was lower in children treated in hospitals with PICUs than in those treated in hospitals with adult ICUs, 0.2% versus 1.0%, respectively [59]. These data support the role of pediatric intensive care capability in higher-risk cases, without implying that ICU admission represents failure of nonoperative management.

Communication between intensive care clinicians, surgeons, radiologists, and nursing staff is essential. Thresholds for concern, transfusion strategies, indications for repeat imaging, and criteria for intervention should be defined clearly. This multidisciplinary coordination supports timely decisions regarding transfusion, repeat imaging, interventional radiology, or surgery when the clinical trajectory changes. In this context, pediatric intensive care should be viewed as a setting for controlled surveillance and rapid escalation in selected higher-risk children, not as evidence that nonoperative management has failed.

### 6.5. Criteria for Progression, De-Escalation, and Discharge

Inpatient management should include not only criteria for escalation, but also criteria for clinical progression and de-escalation of care. As the child stabilizes, monitoring intensity can be reduced gradually according to clinical trajectory. Improvement is suggested by stable or improving vital signs, absence of progressive abdominal symptoms, stable hemoglobin trends, reduced analgesic need, tolerance of oral intake, and increasing comfort with mobilization. Pediatric solid organ injury guidance supports de-escalation of care, transfer from intensive care to a standard ward, reduced laboratory testing, and advancement of activity when these clinical stability criteria are met [2,49].

Discharge planning should be individualized according to injury severity and clinical course. A child may be considered for discharge when hemodynamic stability is sustained, pain can be controlled with simple medication, oral intake is adequate, there is no evidence of active bleeding or untreated complication, and caregivers understand the post-discharge instructions. These instructions should include warning signs requiring urgent reassessment, such as worsening abdominal pain, dizziness, pallor, vomiting, fever, reduced activity, syncope, or new jaundice. Families should also receive guidance regarding temporary activity restriction, the expected pace of recovery, criteria for urgent return, and scheduled outpatient follow-up adapted to injury severity and clinical evolution [60,61].

Outpatient management should be understood as structured post-discharge surveillance rather than simple advice at discharge. It includes scheduled follow-up with the trauma team or pediatric surgery service, reassessment of pain control, activity tolerance, oral intake, and general clinical recovery, reinforcement of activity restriction, and verification that caregivers understand warning signs requiring urgent return. Routine repeat imaging is not necessary for every clinically improving child, but should be considered when new symptoms, persistent pain, fever, jaundice, falling hemoglobin, abnormal laboratory trends, or suspicion of vascular or biliary complications occurs [60,61].

The transition from hospital to home is an important phase of nonoperative management, not a mere administrative endpoint. Clear documentation, structured post-discharge follow-up with the trauma team, and communication with caregivers are essential to ensure continuity and safety after discharge. During hospitalization, clinicians should also provide injury prevention education adapted to the child’s age and mechanism of trauma, including safe transport, helmet use, supervision, sports safety, and recognition of unsafe home or social circumstances when relevant.

## 7. Outcomes, Complications, and Indications for Intervention

A balanced evaluation of nonoperative management requires attention to management outcomes, complications, and the circumstances in which escalation of care becomes necessary. Understanding these aspects is essential for interpreting the benefits and limitations of a nonoperative approach in pediatric hepatic trauma. This section reviews reported outcomes, early and late complications, and indications for interventional or surgical treatment in children managed nonoperatively.

### 7.1. Success Rates and Overall Outcomes of Nonoperative Management

Nonoperative management can provide safe organ-preserving care in appropriately selected children with blunt liver injury, particularly when selection is guided by hemodynamic status, associated injuries, and the availability of structured monitoring. In prospective pediatric blunt liver and spleen injury data, Linnaus et al. reported that nonoperative management failed in 7% of children overall, but failure due to bleeding from liver or spleen injury occurred in only 3% of patients [62]. Earlier pediatric experience also reported that nonoperative management of blunt hepatic and/or splenic trauma was successful in 98% of children [63].

Avoidance of unnecessary surgery remains an important outcome. In a systematic review of high-grade liver trauma, Saqib et al. reported that nonoperative management was successful in 92.4% of high-grade liver injury patients, 194 of 210 cases, close to the overall reported success rate of 95.0% [64]. Pediatric liver-specific data also support this approach. In a 22-year pediatric liver injury experience including 311 children, Landau et al. reported that 93% were successfully treated nonoperatively, with only 4% requiring liver-related laparotomy, an 8% complication rate, and 1% mortality [65].

The prognosis of pediatric blunt liver injury is generally favorable when the child is hemodynamically stable, has no major associated injury requiring laparotomy, and is managed within a structured nonoperative pathway. Available pediatric data indicate low bleeding-related failure and low liver-related laparotomy and mortality rates in appropriately selected cohorts [62,65]. Morbidity is mainly related to increasing transfusion requirement, delayed clinical deterioration, complications requiring interventional radiology or surgery, and associated injuries that alter the expected recovery pathway. Therefore, prognostic assessment should remain dynamic and should integrate initial physiology, response to resuscitation, associated injuries, clinical trajectory, and access to timely escalation.

Successful nonoperative management reduces exposure to laparotomy, postoperative pain, wound complications, adhesions, anesthesia-related burden, and loss of functional hepatic tissue. These benefits should be interpreted within a structured observation-based strategy, not as a passive decision to avoid intervention. These outcomes depend on appropriate patient selection, repeated reassessment, and timely access to interventional radiology or surgery when the clinical trajectory becomes unfavorable.

### 7.2. Length of Hospitalization and Recovery Profile

Hospital length of stay after pediatric liver trauma varies widely and is influenced by several factors, including injury grade, hemodynamic course, associated lesions, transfusion requirement, and institutional discharge protocols. Children with low-grade isolated injuries often recover more quickly and may require relatively short hospitalization, whereas those with high-grade lesions or concomitant trauma frequently need longer observation. However, current pediatric practice has moved away from rigid bed rest and prolonged admission based solely on injury grade. More recent pathways favor discharge according to clinical status, pain control, stability of hemoglobin trends, tolerance of oral intake, and the absence of signs suggesting delayed complications. In a national analysis of 22,153 pediatric blunt spleen and liver injury admissions, Dodgion et al. estimated that use of an abbreviated bed rest protocol could reduce hospitalization by 1.7 days per patient. Similarly, Daodu et al. reported that implementation of an accelerated care pathway in 138 children with blunt solid organ injury reduced mean length of stay from 5.6 to 3.4 days, without differences in operation, embolization, or transfusion requirements [58,60].

After successful nonoperative management, recovery is usually characterized by progressive improvement in pain control, activity tolerance, and oral intake during the first days of hospitalization. Once discharged, recovery usually continues without major interruption, although temporary restriction of strenuous physical activity remains common. The exact duration of post-discharge activity limitation is still variable across centers, reflecting one of the ongoing areas of heterogeneity in pediatric trauma care. Prospective abbreviated bedrest data and subsequent follow-up support the safety of earlier mobilization and recovery-oriented discharge pathways, with no readmissions related to solid organ injury reported in these cohorts [56,57].

From a systems perspective, shorter and more rational hospitalization represents one of the practical benefits of modern nonoperative management. When observation protocols are based on physiologic response rather than on routine prolonged immobilization, hospital resources may be used more efficiently without compromising safety. This is particularly relevant in pediatric centers where trauma beds, intensive care capacity, and imaging resources must be balanced across a wide range of acute conditions [66]. Therefore, hospitalization length should be seen not only as a patient-level outcome, but also as an indicator of the maturity and efficiency of the institutional management pathway.

### 7.3. Early and Late Complications

Complications may occur even after initially successful nonoperative management of pediatric liver trauma. Early complications may include ongoing or delayed hemorrhage, hemodynamic deterioration, transfusion-related issues, and unrecognized associated injuries. In the acute setting, the greatest concern remains persistent bleeding, particularly when accompanied by falling hemoglobin, tachycardia, increasing abdominal distension, or worsening perfusion. In the series by Giss et al., complications occurred in 7 patients, 3.8%, with grade III or IV right lobe liver lacerations and included biloma in 5 patients, hepatic artery pseudoaneurysm with hemobilia in 1 patient, and necrotic gallbladder in 1 patient [67]. All children with complications had fever, persistent or worsening right upper quadrant pain, feeding intolerance, and persistently elevated liver function tests, supporting a low threshold for repeat imaging when the clinical course becomes unfavorable [67].

Later complications may include biliary leakage, biloma formation, hemobilia, intrahepatic or subcapsular collections, infection, pseudoaneurysm formation, delayed rupture, or recurrent bleeding. Biliary complications are uncommon but clinically relevant. Guanà et al. reported biliary complications in 3 children, 2.5%, after pediatric liver trauma, while Kulaylat et al. identified traumatic bile leaks in 11 of 294 children, 3.7%, with blunt hepatic injury [68,69]. These complications may present with fever, abdominal pain, jaundice, gastrointestinal bleeding, persistent inflammatory markers, feeding intolerance, or delayed clinical recovery. Management does not always require surgery and may include image-guided drainage, endoscopic retrograde cholangiopancreatography with sphincterotomy or stenting, selective observation, or operative treatment in selected cases [68,69].

Vascular complications require particular attention because they may be asymptomatic initially but can lead to delayed bleeding. In a pediatric series of post-traumatic liver and splenic pseudoaneurysms, Durkin et al. reviewed 101 children with liver or splenic injuries, including 57 liver injuries, and reported pseudoaneurysm formation in 17 children, 17% [70]. This supports the need for individualized follow-up and repeat imaging when symptoms, high-grade injury, abnormal laboratory trends, or clinical deterioration raise concern for delayed vascular complications. Therefore, the nonoperative pathway should not be equated with minimal care; its safety depends on disciplined surveillance, caregiver education, and timely reassessment after the phase of apparent initial improvement.

The main complications after nonoperative management of pediatric liver injury, together with clinical warning signs, suggested reassessment steps, and possible management options, are summarized in Supplementary Table S1.

### 7.4. Failure of Nonoperative Management

Failure of nonoperative management is generally defined as the need to abandon observation-based management because it no longer provides a safe or sufficient strategy. In pediatric liver trauma, this usually occurs because of ongoing hemorrhage, recurrent hemodynamic instability, increasing transfusion requirement, worsening abdominal findings, or the recognition of associated injuries requiring operative treatment. In the prospective ATOMAC study, Linnaus et al. reported nonoperative management failure in 7% of children with blunt liver and spleen injuries, while failure due to bleeding from the liver or spleen occurred in 3% of patients [62].

Importantly, failure should not be understood as proof that the initial decision was inappropriate. Pediatric trauma care is inherently dynamic, and one of the strengths of nonoperative management is that it allows many children to avoid surgery while preserving the option to intervene promptly if the clinical course worsens. In this sense, successful management depends as much on early identification of failure as on the initial criteria for observation. Worsening physiology, repeated transfusion needs, persistent tachycardia, new peritoneal signs, or radiologic evidence of active bleeding should prompt immediate reassessment of the treatment plan and early multidisciplinary discussion [28].

Predictors of failure remain heterogeneous across studies, partly because definitions differ and because associated injuries often confound interpretation. In practice, concern should increase when clinical evolution becomes discordant with the expected recovery pathway, including persistent or worsening symptoms, unexplained free intraperitoneal fluid, delayed hemoglobin decline, or imaging findings suggesting active bleeding or associated intra-abdominal injury [41,44]. Therefore, observation should be continued only while the child remains clinically stable and can be safely supported within the nonoperative pathway, with prompt escalation when deterioration, persistent bleeding, evolving peritonitis, or another clinically significant complication is suspected.

### 7.5. Indications for Interventional Radiology

Interventional radiology has an increasingly important role in the management of selected pediatric liver injuries, particularly when nonoperative management remains desirable but simple observation is no longer sufficient. Angiography with embolization may be considered in children who have evidence of ongoing arterial bleeding, contrast extravasation on CT, pseudoaneurysm, or recurrent bleeding despite initial hemodynamic stabilization, provided that the child is managed in a center with appropriate expertise and rapid access to the procedure. The overall pediatric evidence base is smaller than in adults, and available data support selective rather than routine use of angioembolization. In a national pediatric blunt solid organ injury analysis, Swendiman et al. reported that angiography or angioembolization was used in a minority of children and that practice varied according to trauma center type, supporting the need for careful patient selection [71].

The value of interventional radiology lies in its ability to control bleeding while avoiding laparotomy in carefully selected cases. It may also be useful for managing certain delayed complications, such as vascular abnormalities or collections requiring image-guided drainage. However, the decision to proceed with embolization must be individualized. Not every radiologic blush mandates intervention, particularly in a child who is clinically stable, and overuse may expose patients to unnecessary procedural risks. In the ATOMAC+ analysis by Naiditch et al., 30 of 1004 children with blunt liver or spleen injury, 3.0%, underwent angiography with or without embolization; among the nine children who underwent liver embolization, four subsequently required operative intervention, although only one required hepatorrhaphy and none required hepatectomy after angioembolization [72]. Therefore, the indication should arise from the combined interpretation of imaging, physiology, laboratory trends, institutional expertise, and the child’s clinical trajectory.

Institutional capability remains crucial. When pediatric-appropriate interventional radiology is unavailable, transfer to a higher-level trauma center should be considered in stable or stabilized patients in whom endovascular treatment is likely to be beneficial. Accordingly, interventional radiology should be viewed as an escalation within the nonoperative pathway, rather than as evidence of failure, when it allows hemorrhage control or management of selected complications without open surgery.

### 7.6. Indications for Surgical Intervention

Despite the success of nonoperative management, surgery remains indispensable in selected pediatric liver trauma cases. The clearest indication is persistent hemodynamic instability despite adequate resuscitation, especially when ongoing intra-abdominal bleeding is strongly suspected. In pediatric intra-abdominal solid organ injury, continued hemodynamic instability remains a major indication for operative intervention [73]. Additional indications include diffuse peritonitis, suspected or confirmed hollow viscus injury, abdominal compartment concerns, or situations in which radiologic or minimally invasive options are unavailable, contraindicated, or unsuccessful. In these cases, operative treatment is not a failure of nonoperative care, but an appropriate escalation within a staged, physiology-guided trauma pathway [8,74].

Infectious complications are uncommon but may require intervention when source control cannot be achieved by less invasive methods. Hepatic abscess, infected biloma, or septic deterioration may occur after high-grade injury or delayed biliary complications. Hsieh et al. reported liver abscess after nonoperative management of blunt liver injury in 6 of 395 patients, 1.5%, with diagnosis occurring at a median of 6 days, range 1–12 days; management included antibiotics and drainage, with no mortality or long-term morbidity reported [75]. In pediatric liver trauma, similar principles apply: antibiotics and image-guided drainage may be appropriate initially, whereas surgery becomes necessary when infection persists, drainage is inadequate, or generalized contamination or sepsis requires definitive source control.

Surgery in pediatric hepatic trauma is usually performed with the goal of hemorrhage control and damage limitation rather than definitive anatomic reconstruction at all costs. Techniques may include packing, control of bleeding surfaces, management of associated injuries, and staged re-exploration when needed. The modern aim is to intervene when necessary, while avoiding excessive operative aggression in a physiologically vulnerable child. This approach aligns with damage control principles and multidisciplinary postoperative support [74,76].

In practical terms, operative escalation should be timely rather than delayed until profound decompensation occurs, particularly in children with persistent instability, ongoing hemorrhage, or physiology compatible with damage-control surgery [77]. Nonoperative management works best when there is continuous readiness to abandon it once it is no longer safe. Therefore, high-quality pediatric liver trauma care is not defined by avoiding surgery at any cost, but by selecting the least invasive strategy compatible with hemostasis, source control, organ preservation, and overall patient safety.

The comparative roles of nonoperative management, interventional radiology, and surgical management in pediatric blunt liver injury are summarized in Supplementary Table S2.

## 8. Future Directions and Perspectives

Although nonoperative management is widely used in hemodynamically stable children with blunt liver injury, several practical aspects remain incompletely standardized. Future studies should focus on clinically actionable criteria, including monitoring intensity, repeat imaging, interventional radiology, discharge timing, activity restriction, and follow-up after discharge. These priorities are important because current practice remains shaped by heterogeneous evidence, institutional resources, and local trauma pathways.

A key direction is the harmonization of clinical definitions and management pathways across pediatric trauma centers. Terms such as hemodynamic instability, transient response to resuscitation, nonoperative management failure, significant hemorrhage, transfusion requirement, and discharge readiness should be defined more consistently. Clearer pediatric criteria would improve comparability across studies and support more reproducible institutional protocols for observation, transfusion support, escalation of care, discharge, and activity restriction.

Further prospective multicenter studies focused specifically on pediatric liver injury are needed. Such studies should clarify how outcomes vary according to injury grade, hemodynamic profile, age, associated trauma burden, transfusion requirement, and institutional resources. They should also better define the incidence, predictors, and optimal management of liver-related complications, including bile leak, biloma, hemobilia, pseudoaneurysm, delayed hemorrhage, and infected collections.

Future research should also refine the role of imaging and interventional radiology. More selective imaging strategies should be evaluated to reduce unnecessary radiation exposure while preserving timely detection of clinically relevant complications. In parallel, clearer thresholds are required for angiography, embolization, and image-guided drainage, especially in children with active bleeding, pseudoaneurysm, hemobilia, bile leak, abnormal laboratory trends, or delayed clinical deterioration.

Post-discharge care also requires stronger evidence. Future studies should evaluate not only survival, avoidance of laparotomy, and complication rates, but also functional recovery, school absence, caregiver burden, psychological impact, return to sports, and time to full reintegration into daily activities. A more individualized recovery model would help align discharge decisions and activity restriction with injury severity, clinical evolution, family understanding, and access to follow-up care.

## 9. Limitations

This review has several limitations. Although the literature search followed a structured strategy, the article was designed as a narrative review rather than as a systematic review or meta-analysis. Therefore, no registered protocol, Preferred Reporting Items for Systematic Reviews and Meta-Analyses (PRISMA) flow methodology, formal risk-of-bias assessment, or quantitative meta-analysis approach was applied. Study selection may therefore have been influenced by narrative judgment, and relevant articles may have been omitted.

The available literature on nonoperative management of pediatric liver injury is heterogeneous, with important differences in study design, patient populations, injury severity, institutional protocols, monitoring strategies, and reported outcomes, making direct comparison difficult. In addition, many studies analyze liver injuries together with other solid organ injuries or include both isolated trauma and polytrauma patients, which may reduce the specificity of liver-focused conclusions. The restriction to English-language publications may also have introduced language bias.

No formal quality assessment tool or grading of the level of evidence was applied to the included studies, which limits the methodological rigor of the review. Therefore, the conclusions should be interpreted as a narrative synthesis of current evidence and clinical experience rather than as a definitive quantitative or comparative effectiveness assessment.

## 10. Conclusions

Nonoperative management is widely used for most hemodynamically stable children with blunt liver injury and can avoid unnecessary laparotomy when applied in appropriately selected patients within a structured trauma system. The available evidence supports a management strategy primarily guided by hemodynamic status, clinical evolution, and associated injuries rather than by imaging grade alone. Its safety depends on serial clinical and laboratory reassessment, timely recognition of complications, and immediate access to escalation strategies such as interventional radiology or surgery when needed.

Important gaps remain regarding the standardization of follow-up, intervention thresholds, repeat imaging, and post-discharge recommendations. Because the current evidence is largely observational and heterogeneous, further pediatric liver-specific studies are needed to refine monitoring pathways, clarify escalation criteria, and optimize recovery. Nonoperative management should therefore be understood as an active organ-preserving strategy requiring continuous reassessment, rather than as a passive alternative to intervention.

## Data Availability

The data that support the findings of this study are available in this article, and further inquiries can be directed to the corresponding author.

## Supplementary Materials

The following supporting information can be downloaded at: https://www.mdpi.com/article/10.3390/medicina62061088/s1, Table S1: Main complications after nonoperative management of pediatric liver injury, warning signs, reassessment steps, and possible management options; Table S2: Comparative role of nonoperative management, interventional radiology, and surgical management in pediatric blunt liver injury.
