# Supplementary material for: Nonoperative Management of Pediatric Liver Injury: Current Evidence, Clinical Indications, and Outcomes
Source: Medicina (Kaunas). 2026 Jun 4;62(6):1088. doi: 10.3390/medicina62061088 (PMC13304151; doi:10.3390/medicina62061088)
Supplement: Supplementary file 1 [file medicina-62-01088-s001.zip › Table S1.pdf]

Table S1. Main complications after nonoperative management of pediatric liver injury, warning signs, reassessment steps, and possible management options

Complication or clinical issue	Clinical warning signs	Suggested reassessment	Possible management options	Key references
**Ongoing or delayed hemorrhage**	Persistent tachycardia, falling hemoglobin, pallor, worsening perfusion, abdominal distension, recurrent hypotension, increasing transfusion requirement	Serial vital signs, repeated abdominal examination, hemoglobin and coagulation trends, repeat contrast-enhanced Computed Tomography (CT) when clinically indicated	Resuscitation, transfusion support, interventional radiology embolization in selected cases, surgery if persistent instability or uncontrolled bleeding occurs	[62,67,73]
**Bile leak or biloma**	Fever, right upper quadrant pain, feeding intolerance, abdominal fullness, jaundice, persistent inflammatory markers, abnormal liver function tests	Liver function tests, inflammatory markers, abdominal ultrasound, contrast-enhanced CT, magnetic resonance cholangiopancreatography or hepatobiliary imaging when available	Observation for small or asymptomatic collections, image-guided drainage, endoscopic retrograde cholangiopancreatography (ERCP) with sphincterotomy or stenting in selected cases, surgery if source control fails	[67–69]
**Hemobilia**	Upper gastrointestinal bleeding, abdominal pain, jaundice, unexplained anemia, delayed hemodynamic changes	Hemoglobin trend, liver function tests, contrast-enhanced CT, CT angiography or catheter angiography when suspected	Selective angiography and embolization, transfusion support if needed, surgery rarely when endovascular control fails	[67,70,72]
**Hepatic artery pseudoaneurysm**	Often asymptomatic initially, delayed abdominal pain, falling hemoglobin, hemobilia, recurrent bleeding, unexplained clinical deterioration	Doppler ultrasound, contrast-enhanced ultrasound (CEUS) when available, contrast-enhanced CT, CT angiography or catheter angiography	Endovascular embolization in selected cases, individualized observation only in carefully selected low-risk situations, surgery if rupture or failed embolization occurs	[67,70,72]
**Infected biloma or hepatic abscess**	Fever, persistent pain, leukocytosis, elevated inflammatory markers, feeding intolerance, delayed recovery, septic deterioration	Complete blood count, inflammatory markers, blood cultures when indicated, ultrasound or contrast-enhanced CT	Antibiotics, image-guided drainage, endoscopic or surgical source control if drainage is inadequate or sepsis persists	[68,69,75]
**Missed hollow viscus or associated intraabdominal injury**	Increasing abdominal pain, guarding, diffuse peritonitis, vomiting, fever, ileus, worsening inflammatory markers, persistent free fluid without clear explanation	Repeated abdominal examination, laboratory reassessment, repeat CT, early surgical consultation	Urgent surgical exploration when perforation, peritonitis, or uncontrolled abdominal contamination is suspected	[4,43,44,73]
**Abdominal compartment concerns or large symptomatic collections**	Progressive abdominal distension, respiratory compromise, oliguria, increasing ventilatory pressure, worsening perfusion	Clinical reassessment, intensive care monitoring, bladder pressure measurement when indicated, imaging to characterize fluid or hematoma burden	Intensive care support, drainage of significant collections when appropriate, decompressive or operative intervention in selected severe cases	[73,76,77]
