# Supplementary material for: Nonoperative Management of Pediatric Liver Injury: Current Evidence, Clinical Indications, and Outcomes
Source: Medicina (Kaunas). 2026 Jun 4;62(6):1088. doi: 10.3390/medicina62061088 (PMC13304151; doi:10.3390/medicina62061088)
Supplement: Supplementary file 1 [file medicina-62-01088-s001.zip › Table S2.pdf]

Table S2. Comparative role of nonoperative management, interventional radiology, and surgical management in pediatric blunt liver injury

Management strategy	Typical clinical context	Main clinical role	Main limitations or risks	Escalation or transition trigger	Key references
**Nonoperative management**	Hemodynamically stable child or sustained responder to initial resuscitation, without diffuse peritonitis or another immediate indication for laparotomy	Organ-preserving strategy based on observation, serial reassessment, laboratory follow-up, imaging when indicated, transfusion support, pain control, mobilization, and discharge planning	Requires institutional capacity for monitoring, imaging, transfusion support, pediatric surgical judgment, and rapid escalation if deterioration occurs	Persistent or recurrent instability, increasing transfusion requirement, active bleeding, worsening abdominal findings, suspected hollow viscus injury, or delayed complications not controlled by observation	[2,8,28,45,48,49]
**Interventional radiology**	Selected patients with active arterial bleeding, contrast extravasation, pseudoaneurysm, hemobilia, recurrent bleeding after stabilization, or collections requiring image-guided drainage	Minimally invasive extension of nonoperative management that may control bleeding or manage selected vascular, biliary, or fluid complications while avoiding laparotomy	Availability varies by center; procedural delay, contrast exposure, vascular access complications, and inappropriate use in clinically stable children without clear indication must be avoided	Persistent bleeding despite stabilization, vascular lesion requiring embolization, symptomatic collection requiring drainage, or failure of observation when endovascular or image-guided control is feasible	[51,70–72]
**Surgical management**	Persistent hemodynamic instability despite resuscitation, diffuse peritonitis, suspected hollow viscus injury, uncontrolled hemorrhage, abdominal compartment concerns, failed embolization, or inadequate source control	Definitive hemorrhage control, treatment of associated injuries, source control, damage control surgery, packing, staged re-exploration, and management of life-threatening complications	Higher invasiveness, anesthesia burden, postoperative pain, wound complications, adhesions, and potential loss of functional hepatic tissue	Immediate need for operative control when physiology, peritonitis, associated injury, or failed minimally invasive management makes continued nonoperative care unsafe	[8,73–77]
